# Endothelial apoptotic activity of angiocidin is dependent on its polyubiquitin binding activity

**DOI:** 10.1038/sj.bjc.6602773

**Published:** 2005-09-13

**Authors:** S Dimitrov, Y Sabherwal, D D Raymond, D Z L'Heureux, Q Lu, G P Tuszynski

**Affiliations:** 1 Department of Neuroscience, Center for Neurovirology, Temple University School of Medicine, 354 Biology Life Sciences Building (015-96), 1900 North 12th Street, Philadelphia, PA 19122, USA

**Keywords:** angiocidin, polyubiquitin, proteosome

## Abstract

We recently cloned the full-length cDNA of a tumour-associated protein. The recombinant protein expressed in bacteria and referred to as angiocidin has potent antitumour activity *in vivo* and *in vitro*. Angiocidin inhibits tumour growth and angiogenesis by inducing apoptosis in endothelial cells. Based on the sequence similarity of angiocidin to S5a, one of the major polyubiquitin recognition proteins in eukaryotic cells, we postulated that the antiendothelial activity of angiocidin could be due in part to its polyubiquitin binding activity. In support of this hypothesis, we show that angiocidin binds polyubiquitin *in vivo* with high affinity and colocalises with ubiquitinated proteins on the surface of endothelial cells. Binding is blocked with an antiubiquitin antibody. Angiocidin treatment of endothelial cells transfected with a proteasome fluorescent reporter protein showed a dose-dependent inhibition of proteasome activity and accumulation of polyubiquitinated proteins. Full-length angiocidin bound polyubiquitin while three angiocidin recombinant proteins whose putative polyubiquitin binding sites were mutated either failed to bind polyubiquitin or had significantly diminished binding activity. The *in vitro* apoptotic activity of these mutants correlated with their polyubiquitin binding activity. These data strongly argue that the apoptotic activity of angiocidin is dependent on its polyubiquitin binding activity.

Ubiquitin is a 76 amino-acid polypeptide that is one of the most highly conserved proteins in nature (Hershko and Ciechanover, 1998). In 1978, ubiquitin was first characterised by Ciehanover and Hershko as a heat stable polypeptide required for the ATP-dependent proteolysis of proteins in rabbit reticulocytes (Ciehanover *et al*, 1978). In the 1980s, the enzyme machinery of this ATP-dependent protein degradation pathway was elucidated by Driscoll and Goldberg (1990) and Rechsteiner *et al* (1993). Today, it is recognised that ubiquitin/proteasome-dependent proteolysis is critically involved in the regulation of many cellular processes such as the cell cycle, differentiation, transcription, antigen presentation and the selective degradation of misfolded and damaged proteins (Jesenberger and Jentsch, 2002). Aberrations of the ubiquitin/proteasome pathway have been thought to play an important role in the pathogenesis of a number of diseases such as Alzheimer’s disease, AIDS, autoimmune disease and cancer. In cancer, proteasome inhibitors have shown antitumour activity in animal models (Adams, 2001) and human cancer trials (Chauhan *et al*, 2005).

We recently cloned the full-length cDNA for this protein and expressed the protein in bacteria. The recombinant protein, referred to as angiocidin, had potent antiangiogenic and antitumour activity (Zhou *et al*, 2004). The recombinant protein induced endothelial apoptosis when added to growth cultures of endothelial cells in full media or when cells were plated on as little as 2 *μ*g of angiocidin adsorbed on wells of a 96-well tissue culture plate.

A blast search of the full-length cDNA of angiocidin revealed that the protein sequence had a high degree of homology with two proteins, S5a (Young *et al*, 1998) and antisecretory factor (Nilsson *et al*, 1992), both of which have identical sequences. However, angiocidin differed from these proteins by three additional amino acids in its carboxyl terminus (G_255_ER_257_). Interestingly, although S5a and antisecretory factor have identical protein sequences, they are reported to have different functions. S5a is an internal polyubiquitin recognition subunit of the 26S proteasome enzyme complex that binds polyubiquitin, the signal that targets proteins for destruction. Antisecretory factor is a secreted protein that inhibits water transport in the bowel induced by cholera toxin. Therefore, S5a/antisecretory factor/angiocidin represent a group of proteins with similar sequence but with diverse functions. Moreover, the difference in structure between S5a/antisecretory factor and angiocidin may additionally cause these proteins to have significantly different conformations. However, as described below both angiocidin, S5a, and antisecretory factor share the same polypeptide sequences that have been shown to mediate binding of polyubiquitinated proteins to S5a (Young *et al*, 1998) and may therefore share common binding interactions despite differences in cellular localization and function.

Based on the identity of the polyubiquitin-binding sequences of angiocidin with S5a, a polyubiquitin recognition protein of the eukaryotic proteasome, we postulated that the antiangiogenic activity of angiocidin could be mediated in part by its polyubiquitin binding activity. In this report, we show that full-length recombinant angiocidin inhibits endothelial proteasome activity, while mutated angiocidin recombinant proteins whose putative polyubiquitin binding sites at positions 216–220 and 290–294 have been replaced with alanines either partially or completely lost their *in vitro* apoptotic activity. These mutant proteins were either unable to bind polyubiquitin or displayed greatly diminished binding activity while angiocidin bound with high affinity. In addition, we show that angiocidin binds to ubiquitinated proteins on the endothelial cell surface and that this binding is blocked with antiubiquitin antibody. These data strongly argue that the apoptotic *in vitro* antiendothelial activity of angiocidin is dependent on its polyubiquitin binding activity. Since many cellular processes such as growth control and cell survival signals depend on a functional proteasome, our data also suggest a novel strategy for the development of anticancer drugs. This strategy proposes to develop polyubiquitin binding peptides and proteins as anticancer therapeutics targeting cells that overexpress ubiquitinated proteins and with a highly active proteasome activity, which include tumour cells and endothelial cells undergoing angiogenesis. These agents would represent a new class of proteasome inhibitors that antagonise the signalling and degradative functions of polyubiquitinated proteins leading to the induction of cellular apoptosis.

## MATERIALS AND METHODS

### Antibodies and reagents

All chemicals were reagent grade unless specified otherwise. Mouse monoclonal anti-his tag antibody was purchased from Qiagen, Valencia, CA, USA. Polyubiquitin was purchased from BioMol, Plymouth Meeting, PA, USA. Rabbit anti-human ubiquitin antibody was purchased from EMD Biosciences, Inc., San Diego, CA, USA. Goat anti-rabbit IgG-Texas red conjugated antibody and Alamar blue were purchased from Biosource, Camarillo, CA, USA. Tissue culture media and serum were purchased from Fisher Scientific, Pittsburgh, PA, USA. Monoclonal and polyclonal antibodies against angiocidin were prepared from purified recombinant protein (Covance, Denver, PA, USA). Fluorescein isothiocyanate was purchased from Pierce Chemical Co., Rockford, IL, USA. PD-10 desalting columns were purchased from Amersham Pharmacia Biotech, Piscataway, NJ, USA. The ImmunoCruz Staining System was purchased from Santa Cruz Biotechnology Inc., Santa Cruz, CA, USA.

### Angiocidin affinity chromatography

Human umbilical vein endothelial (HUVE) cell lysate was prepared from a phosphate-buffered saline (PBS) washed monolayer of 2 × 10^7^ HUVE cells. Monolayers were lysed with 1 ml of 1 × lysis buffer (Cell Signaling, Beverly, MA, USA) containing 1 × concentration of Halt™ protease inhibitor cocktail (Pierce Chemical Co., Rockford, IL, USA) and 1 mM 4-(2-aminoethyl)benzenesulphonyl fluoride (AEBSF). A 1 ml angiocidin-Sepharose column was prepared by coupling 1 mg of angiocidin per ml of CN-bromide activated Sepharose as described in the instructions provided by Amersham Pharmacia, Piscataway, NJ, USA. The column was washed with three column volumes of 10 mM Tris buffer, pH 7.6, containing 10 mM 3-[(3-cholamidopropyl)-dimethylammonio]-1-propanesulphonate (Chaps) detergent, 1 mM CaCl_2_, and 1 mM MgCl_2_ (wash buffer). Half the lysate was passed over the column and the column was then washed with wash buffer. The column was eluted in 10 1-ml fractions with elution buffer (0.1 M Tris buffer, pH 10, containing 10 mM Chaps, 1 mM CaCl_2_, and 1 mM MgCl_2_). Protein peaks were pooled and dialysed against PBS overnight at 4°C. Aliquots of 40 *μ*l were evaluated for ubiquitin by Western blot analysis. As a control, a bovine serum albumin (BSA)-Sepharose column was prepared and HUVE extract applied as described above for angiocidin-Sepharose. No protein peaks were recovered from the BSA-Sepharose column.

### Angiocidin conjugation with fluorescein isothiocyanate (FITC)

An aliquot of 0.250 ml of a 4 mg ml^−1^ solution of angiocidin in PBS was mixed with 0.10 ml of 5% (Na_2_CO_3_), 1 mg of fluorescein isothiocyanate and the volume brought up to 1 ml with distilled water. The reaction was allowed to proceed for 1 h and unreacted fluorescein isothiocyanate was removed by chromatography of the reaction mixture on a PD-10 desalting column equilibrated in PBS. Peak fractions were collected, pooled and the protein concentration determined using the bicinchoninic acid (BCA) protein assay obtained from Pierce.

### Binding assays

The binding of angiocidin and its mutants to polyubiquitin was measured using an enzyme-linked immunosorbent binding assay. Briefly, 0.50 *μ*g of polyubiquitin in 50 *μ*l of 20 mM HEPES buffer, pH 7.4 was dried overnight per well of a 96-well microtiter plate (Nunc MaxiSorp™, Fisher Scientific, Pittsburg, PA, USA). Each well was blocked for 30 min with 200 *μ*l of 1% BSA in PBS, aspirated and washed with 200 *μ*l of PBS. Each well was then incubated for 1.5 h with 100 *μ*l of various concentrations of angiocidin or its mutants in TBST (Tris-buffered saline, pH 7.4, containing 0.05% Tween-20) at room temperature with shaking. Wells were aspirated and washed three times with 250 *μ*l of TBST. Each well was then incubated for 1 h with 100 *μ*l of a 0.70 *μ*g ml^−1^ solution in PBS of biotinylated rabbit antiangiocidin IgG prepared according to the instructions provided with the EZ Link Sulpho-NHS Biotinylation kit obtained from Pierce Chemical Co., Rockford, IL, USA. Wells were then washed as above followed by a 30-min incubation of 100 *μ*l of a 0.05 *μ*g ml^−1^ solution in PBS of streptavidin coupled horseradish peroxidase (Pierce Chemical Co., Rockford, IL, USA). Wells were aspirated, washed three times and developed for 3–15 min with 100 *μ*l of 1-step Ultra TMB (3,3′,5,5′-tetramethylbenzidine) obtained from Pierce Chemical Co., Rockford, IL, USA. Colour development was stopped by the addition of 50 *μ*l of 0.50 M sulphuric acid. The plate was read in an ELISA reader at 450 nm.

### Cell culture

HUVE cells were grown in EBM-2 medium (Cambrex Corporation, East Rutherford, NJ, USA) supplemented with the EBM-2 bullet kit, which contains 2% foetal bovine serum (FBS), and kept in 5% CO_2_ at 37°C.

### Binding of FITC angiocidin and staining of cell surface ubiquitin on HUVE cells

HUVE cells, growing in two well chamber slides, were fixed with 4% paraformaldehyde for 30 min at room temperature. Cells were washed three times with PBS and the endogenous peroxidase activity was blocked for 5 min with Peroxidase Block from the ImmunoCruz Staining System. After three washes with PBS, slides were blocked with 5% horse serum in 0.1% BSA/PBS. For the direct binding studies, cells were then treated in the dark with 2 *μ*g ml^−1^ FITC-angiocidin solution in PBS for 1 h in the presence or absence of 15 *μ*g ml^−1^ antiangiocidin monoclonal antibody, washed three times and the slides coverslipped and viewed by fluorescence microscopy at × 400 magnification. In some experiments, cells were pretreated with 15 *μ*g ml^−1^ of mouse IgG or antiubiquitin antiserum diluted 1–100 for 1 h, washed and then treated with FITC-labelled angiocidin as described above. For the colocalization studies, slides were washed three times after treatment with FITC-labelled angiocidin and then treated with a 1–500 dilution of rabbit antiubiquitin serum for 1 h. After three PBS washes, cells were incubated with a 1–500 dilution of Texas red labelled goat anti-rabbit IgG for 60 min. After washing, slides were mounted and viewed by fluorescence microscopy at × 400 magnification. Controls in which primary antibody was omitted were negative (data not shown).

### Cell transfection

To assess whether proteasome activity is inhibited by treatment with angiocidin, HUVE cells transiently transfected with a fluorescent reporter protein, which is engineered specifically to be degraded by the proteasome, were evaluated in the presence or absence of angiocidin. To measure proteasome activity in HUVE cells, we used the Proteasome Sensor Vector, a reporter vector designed to express the proteasome-sensitive fluorescent protein, ZsProSensor-1 (Clontech, Palo Alto, CA, USA). ZsGreen is a naturally occurring green fluorescent reef coral protein from *Zoanthus* sp. having an excitation maximum of 496 nm and an emission maximum of 506 nm. One day before the transfection, HUVE cells were plated at a density of 1–3 × 10^5^ cells in 2 ml in a 35-mm culture dish (or six-well plate). After overnight incubation when the cells were 50–80% confluent, serum containing EBM-2 medium was replaced with a sterile, serum-free EBM-2 medium.

Cell transfection was performed with FuGene6 (Roche Molecular Biochemicals, Basel, Switzerland). FuGene6 reagent was used at a concentration of 3 *μ*l/1 *μ*g DNA per well for the transfection. At 6–8 h after the transfection reaction, the transfection efficiency was evaluated by measuring the fluorescence of the transfected green fluorescent protein (GFP). In these experiments, transfection efficiency exceeded 30% as assessed with cotransfection experiments using a GFP (pGFP-C1 vector, Clontech, data not shown). Transfected cells were monitored visually using fluorescence microscopy and fluorescence changes were quantitated with a microplate fluorescence reader (Bio-Tek Flx 800, Winooski, VT, USA).

### Construction, expression and purification of his-tagged recombinant angiocidin and its polyubiquitin defective binding mutants

Angiocidin was cloned and expressed as previously described (Zhou *et al*, 2004). Briefly, the full-length cDNA of angiocidin and its mutants were ligated into pTricHis A vector so that the open reading frame was in frame with the 6-histidine tag as previously described (Zhou *et al*, 2004). The constructs were transformed into *Escherichia coli*, and expression of the fusion protein was induced with 1 mM isopropyl-*β*-D thiogalactopyranoside (IPTG). The purified proteins were recovered from Ni-NTA resin and the total protein from bacterial extracts was subjected to SDS–PAGE and Western blotting. The fusion proteins were finally eluted by elution buffer containing a high concentration of imidazole. Endotoxin was completely removed from recombinant proteins by Triton X-114 phase separation as previously described (Aida and Pabst, 1990).

Site-directed mutagenesis was performed to generate the three polyubiquitin defective binding mutants M-1, M-2, M-1-2 using the Quickchange site-mutagenesis kit (Stratagene, LaJolla, CA, USA). Briefly, four primers were designed and used in this experiment. These primers, oriented from the 5′ to 3′ end are as follows:

Primer 1: GTGCTGATCCTGAGGCTGCCGCAGCCGCTCGTGTATCTATGGAAG
Primer 2: CTTCCATAGATACACGAGCGGCTGCGGCAGCCTCAGGATCAGCAC
Primer 3: CTGAGGAAGAGCAGGCTGCTGCTGCCGCACAGATGTCCCTGCAG
Primer 4: CTGCAGGGACATCTGTGCGGCAGCAGCCTGCTCTTCCTCAG.

Primers 1 and 2 were used to generate mutant M-1. Primers 3 and 4 were used to generate mutant M-2. The double-mutant M-1-2 was generated using primer 1 and 2 on the template cDNA obtained from mutant M-1 and primers 3 and 4 were used on the template cDNA obtained from mutant M-2.

### Viability assay

We evaluated the effect of angiocidin on cell viability using the Alamar blue assay. In this assay, 10 *μ*l Alamar blue, a metabolizable dye, was added to a 100 *μ*l of media in a 96-well plate containing 15 000 cells per well. After 2–3 h, the number of viable cells in the plate was proportional to the difference in absorbance at 562 and 595 nm as read in a microtitre plate reader.

### Western blots

Proteins were analysed under reducing conditions by sodium dodecyl sulphate–polyacrylamide gel electrophoresis (SDS–PAGE), and the gel electroblotted onto a polyvinylidene fluoride (PVDF) membrane. Gels (12% polyacrylamide) were used in the study and were purchased from GeneMate ISC BioExpress, Kaysville, UT, USA. The membrane was developed using the chemiluminescence (ECL) system (Amersham, Arlington Heights, IL, USA).

## RESULTS

### Polyubiquitin binding activity of angiocidin

**Figure 1:**
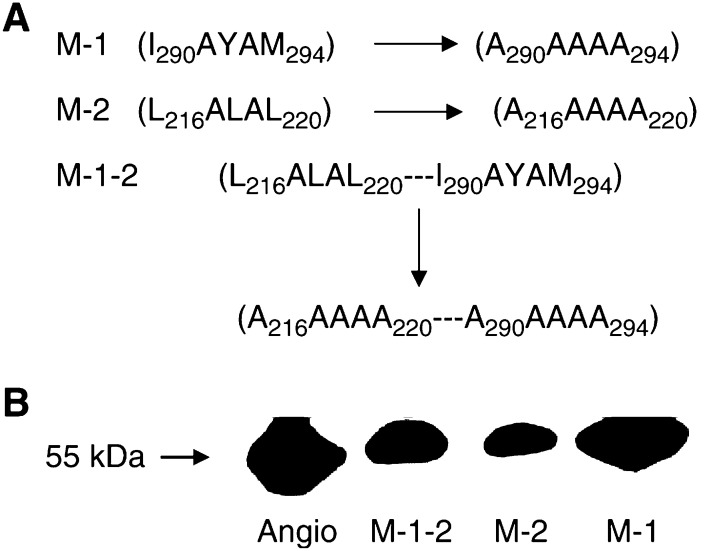
Polyubiquitin deficient binding mutants. (**A**) Description of altered sequences in the mutated angiocidin recombinant proteins. (**B**) Western blot of recombinant angiocidin. Proteins were probed with a 1 *μ*g ml^−1^ polyclonal antiangiocidin IgG as previously described (Zhou *et al*, 2004).

Based on the fact that angiocidin contains peptide domains homologous to the polyubiquitin binding domain of S5a, one of the major polyubiquitin recognition subunits of the proteasome, we speculated that the antitumour activity and apoptotic activity of angiocidin may in part be mediated by peptide regions of angiocidin that bind polyubiquitin. Thus far we have shown that the thrombospondin-1 binding domain of angiocidin partially regulates its apoptotic activity *in vitro* while abolishing its *in vivo* antitumour activity (Zhou *et al*, 2004). However, we could not render the molecule completely inactive *in vitro* by mutating the TSP-1 binding site alone. Therefore, we sought to find other domains of the molecule that mediate its apoptotic activity. We reasoned that the polyubiquitin binding domains of angiocidin could render it a competitive inhibitor of the cellular proteasome by complexing with polyubiquitinated proteins both on the cell surface as well as in the cytoplasm. These proteins complexed with angiocidin might then be prevented from degradation by the endogenous cellular proteasome. Since many cellular proteins that regulate apoptosis are degraded by the proteasome, inhibition of the proteasome would be expected to result in the induction of apoptosis (Jesenberger and Jentsch, 2002). We expressed three his-tagged recombinant angiocidin molecules that had their putative polyubiquitin binding domains substituted for alanines as was performed for S5a (Young *et al*, 1998). For S5a at least two polyubiquitin binding sites have been reported to reside in an alpha helical region of the protein near the carboxyl terminus of the molecule (Young *et al*, 1998). These sites have been reported to differ in affinity for polyubiquitin. Therefore, we created the following mutants of angiocidin based on the polyubiquitin binding structure of S5a: M-1 has I_290_AYAM_294_, a low-affinity polyubiquitin-binding site substituted with alanines. M-2 has L_216_ALAL_220_, a high-affinity polyubiquitin-binding site substituted with alanines. M-1-2 has both sites substituted with alanines (Figure 1). The purified recombinant angiocidin and its alanine substitution mutants contained a 55 kDa polypeptide that crossreacted with an antiangiocidin monoclonal antibody, as revealed by Western blot analysis (Figure 1B).

**Figure 2:**
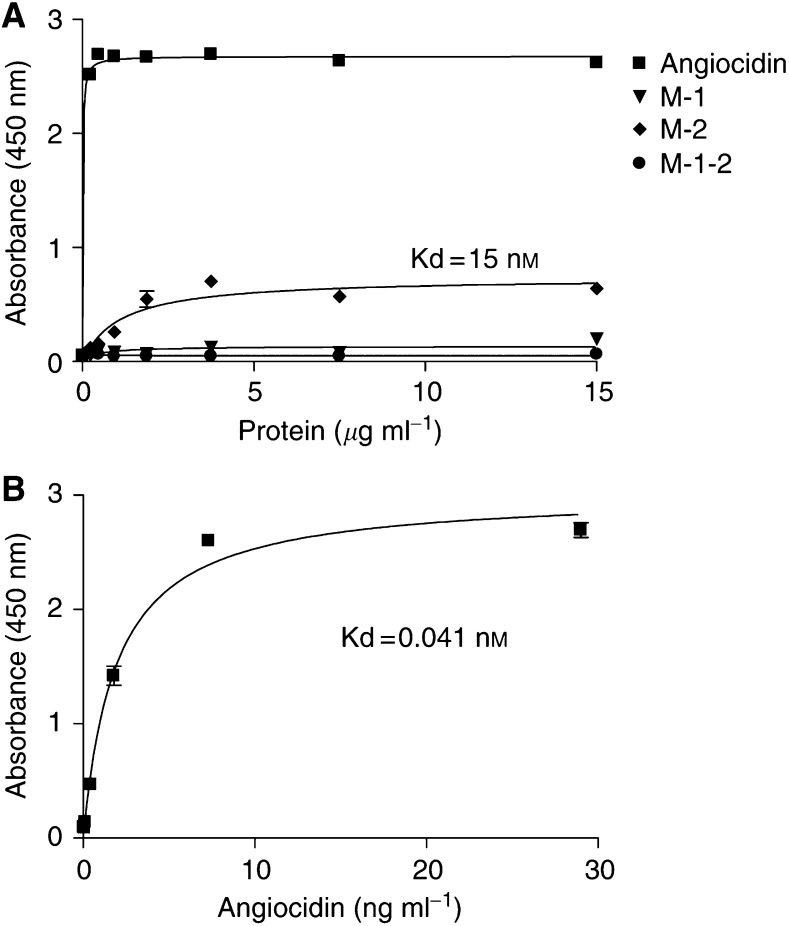
Binding of angiocidin and its polyubiquitin deficient mutants to polyubiquitin. Wells of a 96-well plate were coated with 0.5 *μ*g of polyubiquitin and binding was performed as described in the Materials and Methods section. Lines were fitted to a rectangular hyperbola and Kd and maximal bound angiocidin were calculated using GraphPad Prizm software (San Diego, CA, USA). (**A**) Binding at a high protein concentration range. (**B**) Angiocidin titrated down to a lower range allowing for the determination of a Kd value. Error bars represent the standard deviation. The experiments shown are representative of at least two separate experiments.

To confirm that mutants M-1-2, M-1, and M-2 lost their polyubiquitin binding activity, we tested their binding activity in an ELISA binding assay in which polyubiquitin was adsorbed on a microtitre plate. Bound angiocidin or its mutants was detected with a biotinylated-labelled rabbit antiangiocidin antibody raised against recombinant angiocidin (Figure 2). Polyubiquitin was purchased commercially and contains a range of ubiquitin polymers from 1 to 50 kDa. The data in Figure 2 show that mutant M-1, and M-1-2 completely lost their polyubiquitin binding activity while angiocidin and M-2 bound saturably with a Kd of 0.041 and 15 nM, respectively. Although M-2 bound saturably, its Kd was over 300 fold higher than that of angiocidin and the maximal bound protein was four-fold lower than that for angiocidin (compare Figure 2A with Figure 2B).

### *In vitro* apoptotic activity of angiocidin is dependent on its polyubiquitin binding activity

**Figure 3:**
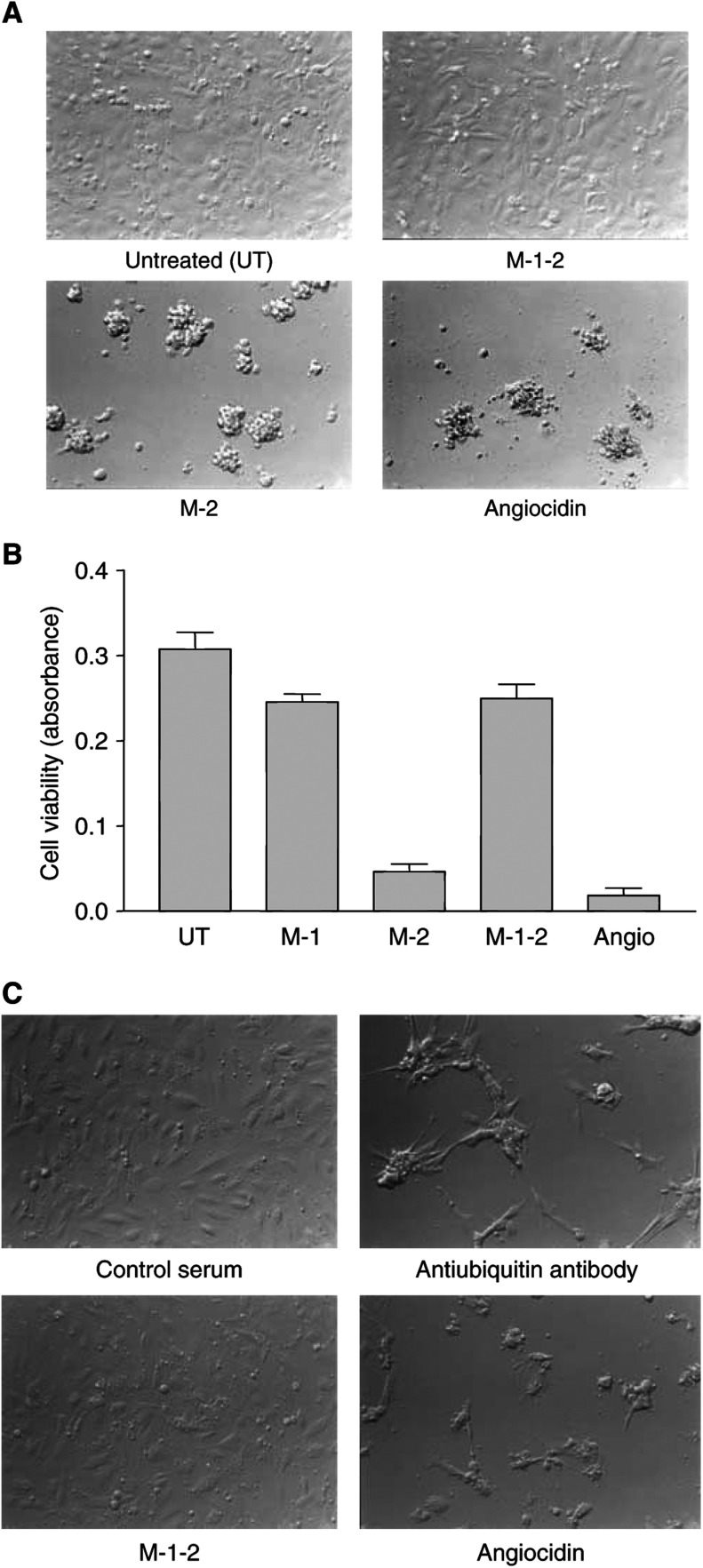
Effect of angiocidin, its mutants and antiubiquitin antibody on the viability of HUVE cells. Cells were plated on 96-well microtitre plates coated with either 1 *μ*g of angiocidin or its mutants. Cells were cultured overnight and viability measured using the Alamar blue assay as previously described (Zhou *et al*, 2004). (**A**) Cells photographed using Hoffman interference microscopy at a magnification of × 100. (**B**) Viability measurements using the Alamar blue assay. The experiment is representative and shows the mean of three replicates and error bars represent the standard error of the mean. (**C**) Effect of antiubiquitin antibody on the viability of HUVE cells. HUVE cells were grown for 24 h on 96-well plates coated with 1 *μ*g of angiocidin or M-1-2 or treated with 10 *μ*l of control serum or rabbit antiubiquitin antibody. Cells were photographed using Hoffman interference microscopy at a magnification of × 200.

Angiocidin and its polyubiquitin-binding mutants were compared for their endothelial cell apoptotic activity using the Alamar blue viability assay. We found that mutants M-1, containing residues I_290_ AYAM_294_ substituted by alanines, and M-1-2, containing residues I_290_ AYAM_294_ and L_216_ALAL_220_ substituted by alanines, lost their apoptotic inducing activity as well as their polyubiquitin binding activity. However, M-2, containing L_216_ALAL_220_ substituted with alanines, still bound polyubiquitin with a favourable Kd of 1.52 nM, while displaying four-fold lower maximal binding than angiocidin. In contrast to the other mutants that completely lost their polyubiquitin binding activity and their apoptotic activity, M-2 had a two-fold diminished apoptotic activity as compared to angiocidin (Figure 3B). Many more cells although rounded were still viable in the M-2 treatment group (Figure 3A). Cells were treated overnight with angiocidin and its mutants. These results indicate that the extent to which angiocidin binds polyubiquitin correlates with its *in vitro* apoptotic activity.

### Antibody to ubiquitin mimics the apoptotic activity of angiocidin

To provide additional evidence that angiocidin partially exerts its apoptotic activity on HUVE cells by binding ubiquitinated proteins, we compared the activity of angiocidin with that of an antibody against ubiquitin (Figure 3C). Cells were treated overnight. Both angiocidin and the antiubiquitin antibody induced cell death approximately to the same extent (as approximated by trypan blue dye exclusion), suggesting that both proteins interact with ubiquitinated proteins either on the cell surface or internally to induce cell death.

### Angiocidin binds ubiquitinated proteins *in vivo* and *in vitro*

**Figure 4:**
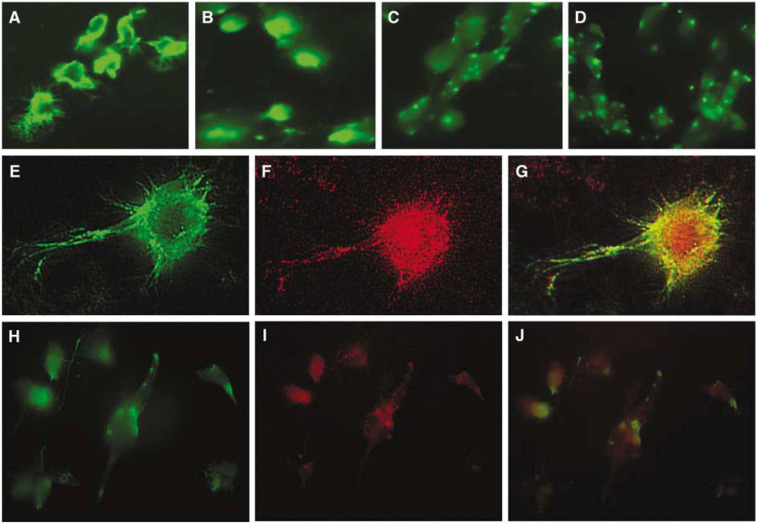
Angiocidin colocalises with ubiquitinated proteins in HUVE cells. HUVE cells, growing in glass chamber slides, were fixed with 4% paraformaldehyde and treated with 2 *μ*g ml^−1^ FITC-angiocidin and costained with antiubiquitin antibody, as described in Material and Methods. After washing, slides were mounted and viewed by fluorescence microscopy at × 400 magnification. In panels A–D, cells were only treated with 2 *μ*g ml^−1^ FITC-angiocidin or 2 *μ*g ml^−1^ FITC-angiocidin in the presence of 15 *μ*g ml^−1^ of control mouse IgG, or antiangiocidin mouse monoclonal IgG, or a 1–100 dilution of antiubiquitin antiserum. In panels E–J, slides were treated with 2 *μ*g ml^−1^ FITC-angiocidin and costained with antiubiquitin antibody. Panels E and H show FITC-angiocidin staining, panels F and I show antiubiquitin staining, and panels G and J are composites of FITC-angiocidin and antiubiquitin staining showing yellow areas of colocalization of angiocidin and antiubiquitin stained areas. Controls in which primary antibody was omitted were negative (data not shown).

To evaluate the interaction of angiocidin with ubiquitinated proteins *in vivo*, we investigated the cell surface interaction of HUVE cells with angiocidin and ubiquitin. Angiocidin was derivatised with the fluorescent marker FITC. Derivatised angiocidin did not lose any of its endothelial apoptotic activity as compared to uncoupled angiocidin (data not shown). We found that HUVE cells fixed with paraformaldehyde bound FITC-labelled angiocidin (Figure 4A, E and H). In general, FITC-labelled angiocidin appeared to localise to filopodia, the periphery of the cell, and in punctate spots showing a nonuniform distribution. In the presence of a monoclonal antibody to angiocidin and a polyclonal antibody to ubiquitin, the binding was significantly inhibited (compare A with C and D of Figure 4). In contrast, control IgG did not significantly inhibit binding of FITC-angiocidin (compare A with B of Figure 4). Angiocidin and ubiquitin both localised to filopodia and to punctate patches distributed near the periphery of the cells (Figure 4 E–J). Colocalization was most apparent in the areas showing strong binding such as in punctate patches, cell periphery and filopodia (Figure 4G and J).

**Figure 5:**
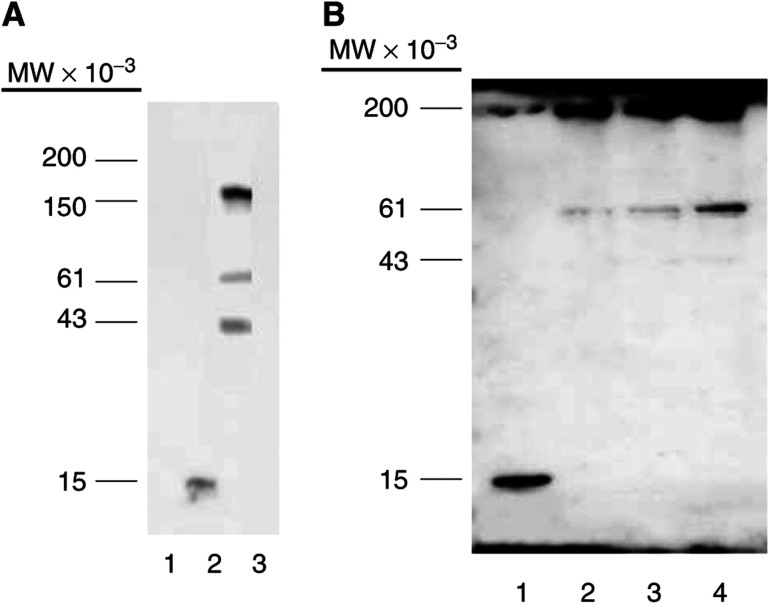
Angiocidin binds HUVE polyubiquitinated proteins as well as inducing HUVE cells to accumulate polyubiquitinated proteins. In panel A, HUVE cell eluates obtained from an angiocidin- or BSA-Sepharose column were analysed on SDS–PAGE under reducing conditions and Western blotted for polyubiquitinated proteins using purified rabbit antipolyubiquitin antiserum at a dilution of 1–500. The blot was developed by enhanced chemiluminescence. Approximately 5 *μ*g of column eluate and 1 *μ*g of purified ubiquitin were analysed. Lane 1, purified ubiquitin; Lane 2, angiocidin affinity column eluate; Lane 3, BSA column eluate. In panel B, HUVE cells grown in 24-well plates were treated with either buffer or with 100 *μ*g ml^−1^ angiocidin for 5 or 24 h, harvested and Western blotted for polyubiquitin as described in panel A. Lane 1, purified ubiquitin; Lane 2, buffer treated cells; Lane 3, cells treated with angiocidin for 6 h; Lane 4, cells treated with angiocidin for 24 h.

To identify ubiquitinated proteins recognised by angiocidin, HUVE cell detergent extracts were chromatographed on an angiocidin affinity column and the column eluates were Western blotted with antiubiquitin antibody. We found three major ubiquitinated proteins from HUVE cells that bound to the column. These proteins ranged in molecular weight from 40 to 200 kDa (Figure 5A). We have tentatively identified one of the bands isolated by angiocidin affinity chromatography using MALDI-TOF peptide fingerprinting. The 150 kDa band gave rise to peptide fragments matching those of tissue transglutaminase 2. We are currently investigating the significance of this finding in relationship to mechanisms of angiogenesis. Tissue transglutaminase is thought to mediate angiogenesis by crosslinking the extracellular matrix (Haroon *et al*, 1999).

### Angiocidin induces HUVE cells to accumulate polyubiquitinated proteins and inhibits their proteasome activity

To further provide evidence that angiocidin exerts its apoptotic activity by interfering with endogenous ubiquitin binding proteins, we evaluated the level of protein ubiquination in HUVE cells treated with angiocidin. We found that cells treated with angiocidin or plated on angiocidin-coated tissue culture plates contained significantly higher levels of ubiquitinated proteins than cells plated on plastic or treated with buffer as revealed by Western blot analysis (Figure 5B). Three ubiquitinated proteins having molecular weights of approximately 200 000, 61 000, and 43 000 Da increased in angiocidin-treated cells as compared to controls (Figure 5B). Similar proteins were found to bind to an angiocidin affinity column (Figure 5A).

**Figure 6:**
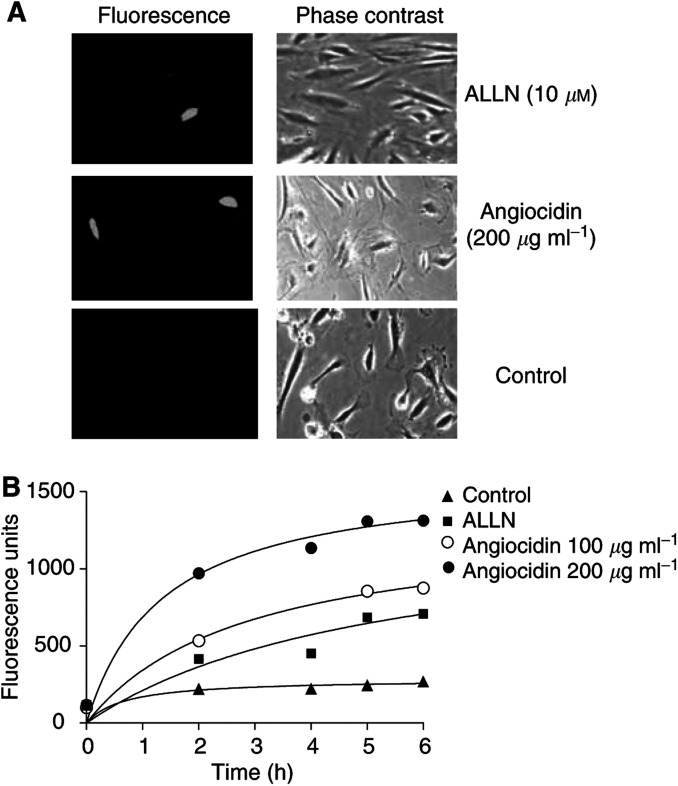
Angiocidin inhibits proteasome activity of HUVE cells. HUVE cells were transiently transfected with a proteasome reporter plasmid encoding ZsGreen, a fluorescent protein degraded by the proteasome, and plated in 96-well culture plates in serum-containing media for quantitative fluorescence measurements or in six-well plates for photography. After cells attached, they were either treated with buffer (control), angiocidin (100–200 *μ*g ml^−1^), or ALLN (10 *μ*g ml^−1^), a known proteasome inhibitor. At various times, fluorescence intensity was measured (**B**) and the cells were photographed after 3 h as viewed by fluorescence and phase microscopy at × 200 magnification (**A**).

To provide additional data that components of the cellular ubiquitin recognition system are inhibited by angiocidin, we assayed proteasome activity of cells treated with angiocidin. HUVE cells were transiently transfected with a fluorescent reporter protein that is specifically degraded by the proteasome and the activity of the reporter was measured in the presence or absence of angiocidin. To measure proteasome activity in HUVE cells, we used the Proteasome Sensor Vector, a reporter vector designed to express the proteasome-sensitive fluorescent protein, ZsProSensor-1 (Clontech, Palo Alto, CA, USA). This protein is a C-terminal fusion of ZsGreen with the mouse ornithine decarboxylase degradation domain (amino acids 410–461). ZsGreen is a naturally occurring green fluorescent coral reef protein from *Zoanthus* sp. with an excitation maximum of 496 nm and an emission maximum of 506 nm. The addition of the mouse ornithine decarboxylase degradation domain targets the protein for rapid degradation by the proteasome. In this assay, transfected cells internally accumulate the fluorescent undegraded protein and fluoresce green when the proteasome is inhibited. As a positive control, cells were treated for 3–5 h with 10 *μ*M of a known proteasome inhibitor ALLN (*N*-acetyl-Leu-Leu-Norleucinal). This peptide–aldehyde adduct is freely cell permeable and inhibits the neutral cysteine protease activity of the proteasome. In these experiments, transfection efficiency exceeded 30% as assessed with cotransfection experiments using a GFP (pGFP-C1 vector, Clontech, data not shown). Transfected cells were monitored visually using fluorescence microscopy and fluorescence changes were quantitated with a microplate fluorescence reader. We observed a dose-dependent increase in fluorescence when HUVE cells growing in serum-containing media were treated with increasing concentrations of angiocidin as quantitated with a fluorescent ELISA reader (Figure 6B) and visualised by fluorescence microscopy (Figure 6A). These results provide strong evidence in support of the hypothesis that angiocidin inhibits cellular proteasome activity.

## DISCUSSION

We previously isolated a protein from lung tumour extracts by peptide affinity chromatography using the type 1 TSP-1 repeat peptide CSVTCG (Tuszynski *et al*, 1993). The protein was cloned from a prostate cell cDNA library and the full-length cDNA was nearly identical to that reported for S5a, a ubiquitin recognition subunit of the 19S proteasome subunit and antisecretory factor, a secreted protein that regulates water secretion by the large bowel (Johansson *et al*, 1997). We have named our cloned protein angiocidin due to its *in vitro* antiangiogenic activity (1). Like S5a, angiocidin contains a polyubiquitin binding sequence and like antisecretory factor angiocidin is secreted into the circulation. We have also previously shown that recombinant angiocidin has potent antitumour as well as endothelial cell apoptotic activities, and that the antitumour and endothelial apoptotic activities of angiocidin depend partially on its TSP-1 binding activity (Zhou *et al*, 2004). Furthermore, in these studies, we also found that recombinant angiocidin injected into tumour-bearing mice bound to the tumour stroma and induced massive tumour cell death.

It is well recognised that protein degradation mediated by cellular proteasome activity contributes to the malignant phenotype. Recognition of these polyubiquitinated proteins both on the cell surface and internally contributes to cellular proteasome activity. Therefore, we postulated that putative polyubiquitin binding domains of angiocidin may contribute to its ability to induce endothelial cell apoptosis. Two sequences present in angiocidin are identical to the sequences in S5a that have been shown to bind polyubiquitin (Young *et al*, 1998). These two sequences were mutated by substituting alanines for amino acids present in residues 216–220 and 290–294. Three mutant angiocidin recombinant proteins were expressed (Figure 1). Since residues I_290_–M_294_ of S5a have been reported to contain a low-affinity polyubiquitin-binding site (Young *et al*, 1998), mutant M-1 angiocidin containing I_290_–M_294_ substituted with A_290_ AAAA_294_ was expressed and purified. Additionally, since residues L_216_–l_220_ of S5a have also been reported to contain a high-affinity polyubiquitin-binding site (Young *et al*, 1998), we also elected to express mutant M-2 containing L_216_–L_220_ substituted with A_216_AAAA_220_. Finally, mutant M-1-2 having both polyubiquitin binding sites substituted with alanines was also expressed. We found that all the polyubiquitin binding mutants displayed either no or diminished binding to polyubiquitin immobilised on microtitre dishes. Angiocidin bound with a Kd of 0.041 nM, while M-2 bound with a Kd of 15 nM and M-1 and M-1-2 showed no detectable binding (Figure 2).

The functional consequence of mutating the polyubiquitin binding site of angiocidin was investigated using the Alamar blue viability assay. In our previous study (Zhou *et al*, 2004), we used this assay to show that angiocidin induces endothelial cell apoptosis. As little as 1–2 *μ*g of angiocidin adsorbed on the surface of a 96-well-microtitre dish caused cells to round and undergo apoptosis in as short a time as 5 h. When we tested our polyubiquitin binding-deficient mutants, we found that mutants M-1 and M-1-2 completely lost their apoptotic activity, while M-2 although still active displayed at least 50% less activity than angiocidin (Figure 3). The *in vitro* apoptotic activity of angiocidin appeared to directly correlate with its capacity to bind polyubiquitin. Mutants M-1 and M-1-2 that bound no polyubiquitin displayed no *in vitro* HUVE cell apoptotic activity, while mutant M-2 that still bound polyubiquitin had a two-fold lower apoptotic activity as compared to angiocidin. Furthermore, in the absence of a consideration of possible protein conformational changes induced by substituting alanines in the active polyubiquitin binding sites of angiocidin, these results also suggest that residues I_290_–M_294_ play a more important role than residues L_216_–L_220_ in the binding of polyubiquitin and the induction of endothelial cell apoptosis.

Our data regarding the polyubiquitin deficient binding mutants suggest that angiocidin induces cell apoptosis in HUVE cells by inhibiting cellular proteasome activity by interaction with the cellular polyubiquitin recognition system. In support of this hypothesis, we show that angiocidin-treated HUVE cells accumulate ubiquitinated proteins as seen by Western blot analysis of total cell lysates prepared from cells treated with angiocidin for increasing lengths of time (Figure 5B). The functional consequence of this increase in ubiquitin protein accumulation was evaluated using a proteasome reporter assay. We found that both angiocidin and a known proteasome inhibitor, *N*-acetyl-leucinyl-leucinyl-norleucinal (ALLN), caused a time-dependent increase in the fluorescence of HUVE cells transfected with the proteasome reporter protein (Figure 6A and B). These results strongly suggest that angiocidin inhibits cellular proteasome activity.

To further provide evidence that angiocidin induces apoptosis in HUVE cells by antagonizing the cellular ubiquitin recognition system of the proteasome, we performed experiments designed to show that angiocidin binds to ubiquitinated proteins in HUVE cells. In the first experiment, HUVE cell lysates were fractionated by angiocidin affinity chromatography (Figure 5A). We detected at least three ubiquitinated protein bands ranging in molecular weight from 150 to 43 kDa. These proteins are currently being characterised by mass spectral analysis. Preliminary mass spectral analysis of one of these protein bands indicates homology with tissue transglutaminase. The significance of this observation is currently under investigation. Consistent with these results we also found that angiocidin bound unpermeabilised HUVE cells and colocalised with surface-expressed ubiquitin in a peripheral as well as a punctuate pattern of colocalization (Figure 4). Colocalization was also observed on filopodia. We were able to block FITC-angiocidin binding with an antibody to angiocidin and ubiquitin, indicating that the angiocidin binding was specific and that it occurred through cell surface ubiquitin. These results strongly suggest that angiocidin binds to cell surface ubiquitinated HUVE cell proteins. Consistent with these results is our observation that an antibody to ubiquitin was found to mimic the apoptotic activity of angiocidin (Figure 3). Like angiocidin, we postulate that antiubiquitin antibody must bind to HUVE cell surface proteins and induce cell death. Taken together these results are consistent with the concept that cell-surface ubiquitinated proteins mediate HUVE cell apoptosis.

The major function of protein ubiquitination is the signalling of protein degradation associated with cellular house-keeping functions such as the elimination of damaged proteins. However, it has become clear in recent years that post-translational modification of proteins with ubiquitin is a major cellular regulatory mechanism. Both the turnover and activity of many cell surface receptors are regulated by ubiquitination. A notable example is epidermal growth factor receptor (EGFR). This receptor or its splice variants are upregulated in many malignant tumours such as human glioma and center carcinoma. Upon ligand activation, EGFR is rapidly polyubiquitinated by specific adaptor proteins (Schmidt *et al*, 2003). Our results confirm the presence of numerous ubiquitinated proteins on the surface of endothelial cells as revealed by immunostaining of paraformaldehyde-fixed cells. In this report we show that fluorescence-conjugated angiocidin binds to the surface of endothelial cells and colocalises with ubiquitinated proteins. We speculate that angiocidin binds to critical receptors important in cell adhesion and growth perhaps through regions of these proteins derivatised with ubiquitin. It is conceivable that these proteins could be adhesion receptors that mediate critical cellular adhesive events important in angiogenesis and survival. Angiocidin could bind these proteins either through domains that become polyubiquitinated during ligand binding such has been observed for EGFR or through other domains that interact with angiocidin. Angiocidin-ligated receptors may be inhibited from turnover and subsequently lose their ligand binding activity. These possibilities are currently being investigated.

Our results do not exclude the importance of matrix binding domains of angiocidin in promoting its antitumour activity such as the TSP-1 domain identified in our previous study (Zhou *et al*, 2004). We postulate that these matrix binding domains of angiocidin help localise angiocidin to the tumour environment that is rich in matrix proteins such as TSP-1 and collagen. Angiocidin is then able to interact with cell surface ubiquitinated proteins and exert its inhibitory effect on angiogenesis.

The implications of these studies regarding angiocidin as a potential anticancer therapeutic are considerable. Angiocidin could be used to target tumour cells and tumour vasculature by binding to specific polyubiquitinated proteins expressed on tumour and tumour vasculature. In fact, our preliminary studies have shown that angiocidin accumulates on the tumour vasculature and tumour stroma of mice bearing Lewis Lung carcinoma (Zhou *et al*, 2004). Angiocidin would then exert its antiangiogenesis activity.

Finally, in a much broader context, our results suggest that targeting ubiquitinated proteins may represent a new paradigm for the development of novel antiangiogenic cancer therapeutics. Ubiquitin protein antagonists may not only inhibit cellular proteasome activity but may also target surface proteins important for cell growth and angiogenesis. Therefore, the identification of small molecule ubiquitin binding compounds may represent an attractive strategy for anticancer drug discovery.
